# Synergistic Action of Immunotherapy and Nanotherapy against Cancer Patients Infected with SARS-CoV-2 and the Use of Artificial Intelligence

**DOI:** 10.3390/cancers14010213

**Published:** 2022-01-02

**Authors:** Tanvi Gupta, Tilahun Ayane Debele, Yu-Feng Wei, Anish Gupta, Mohd Murtaza, Wen-Pin Su

**Affiliations:** 1 Institute of Clinical Medicine, College of Medicine, National Cheng Kung University, Tainan 704, Taiwan; s98097021@gs.ncku.edu.tw; 2 Department of Biomedical, Chemical & Environmental Engineering, College of Engineering and Applied Science (CEAS), University of Cincinnati, Cincinnati, OH 45221, USA; debeleta@ucmail.uc.edu; 3 Department of Internal Medicine, School of Medicine for International Students, College of Medicine, E-Da Cancer Hospital, I-Shou University, Kaohsiung 824, Taiwan; ed102746@edah.org.tw; 4 Devscope IT, First Floor, 40A/B Gandhi Nagar, Jammu 180001, India; gupta30anish@gmail.com; 5 Microbial Biotechnology Division, CSIR-Indian Institute of Integrative Medicine, Jammu 180012, India; murtazarazivi1370@gmail.com; 6 Departments of Oncology and Internal Medicine, National Cheng Kung University Hospital, College of Medicine, National Cheng Kung University, Tainan 704, Taiwan; 7 Center of Applied Nanomedicine, National Cheng Kung University, Tainan 704, Taiwan

**Keywords:** cancer, immunotherapy, nanotherapy, artificial intelligence

## Abstract

**Simple Summary:**

Cancer patients infected with SARS-CoV-2 are the most vulnerable and susceptible due to their poor immune response. Combination of nano-carriers based vaccines and immune checkpoint inhibitors could counter-act the infection. With the access of real- time data from the patients, it will be easy to predict and analyze through artificial intelligence (AI); the severity of risk at an early stage and physicians can alter or modify their treatment regime. This new ideology by amalgamation of therapies and machine learning will be best for the clinicians and physicians to provide the best treatment for their patients.

**Abstract:**

Since 2019, the SARS-CoV-2 pandemic has caused a huge chaos throughout the world and the major threat has been possessed by the immune-compromised individuals involving the cancer patients; their weakened immune response makes them vulnerable and susceptible to the virus. The oncologists as well as their patients are facing many problems for their treatment sessions as they need to postpone their surgery, chemotherapy, or radiotherapy. The approach that could be adopted especially for the cancer patients is the amalgamation of immunotherapy and nanotherapy which can reduce the burden on the healthcare at this peak time of the infection. There is also a need to predict or analyze the data of cancer patients who are at a severe risk of being exposed to an infection in order to reduce the mortality rate. The use of artificial intelligence (AI) could be incorporated where the real time data will be available to the physicians according to the different patient’s clinical characteristics and their past treatments. With this data, it will become easier for them to modify or replace the treatment to increase the efficacy against the infection. The combination of an immunotherapy and nanotherapy will be targeted to treat the cancer patients diagnosed with SARS-CoV-2 and the AI will act as icing on the cake to monitor, predict and analyze the data of the patients to improve the treatment regime for the most vulnerable patients.

## 1. Introduction

By the end of year 2019, an outbreak of novel coronavirus occurred in the Hubei province, Wuhan, China and later in March, 2020 it was declared as pandemic all across the world by World Health Organization (WHO) [1]. This pandemic was caused due to severe acute respiratory syndrome associated coronavirus-2 (SARS-CoV-2), commonly known as COVID-19 [2,3]. These coronaviruses belong to the family of enveloped, positive sense, single strand RNA virus that can infect birds as well as mammals. By the December 2021, 278 million confirmed cases, and nearly 5.39 million deaths were reported across the globe. The topmost leading countries with high number of cases are United States with over 52 million cases and India with over 34.7 million cases, as reported by COVID-19 Data Repository by the Center for Systems Science and Engineering (CSSE) at Johns Hopkins University (JHU CSSE COVID-19 Data).

The major symptoms of COVID-19 include fever, sore muscles, fatigue, headaches, septum production, hemoptysis and breathlessness [4,5] SARS-CoV-2 can be transmitted to an individual from an infected person with or without any symptoms with a reproduction number (Ro) value. Initially, the Ro was in between 2.2 and 2.7, later Ro value increased 4.7 to 6.6 which makes the doubling time of 2.4 days [6,7,8]. Many studies have revealed that higher rate of transmission has been observed for COVID-19 compared to Middle East respiratory syndrome (MERS) and severe acute respiratory syndrome (SARS) [6]. Throughout the world, travel has played a major contributing factor for the transmission of the virus to each and every corner [9,10].

**Figure 1:**
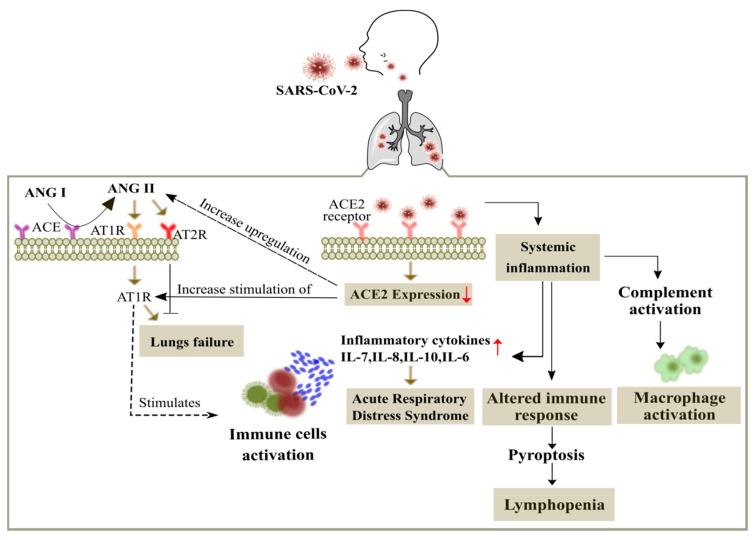
Overview of the SARS-CoV-2 in the cancer patients. The entry of SARS-CoV-2 is mediated by binding of the spike protein to the angiotensin-converting enzyme II (ACE2) receptor (abundantly expressed on alveolar epithelial cells) with a subsequent membrane fusion leading to the down regulation of the ACE2 receptor. This in turn leads to the activation of angiotensin type 1 receptor (AT1R) through up regulation of angiotensin (ANG II). ANG II is also produced from ANG I through the renin-angiotensin system regulated by the angiotensin-converting enzyme (ACE). Up regulation of ANG II leads to respiratory failure and ANG II interferes with the adaptive immunity by activation of immune cells and increases the production of inflammatory cytokines to promote acute respiratory distress syndrome (ARDS). Viral infected patients also show widespread complement activation that leads to accumulation of macrophages, along with lymphopenia as an indicator of the seriousness and hospitalization in the infected patients.

COVID-19 report showed that the patients with poor immune response were worst affected and they were prone towards the pathogens. The patients suffering from multiple diseases/disorders have more chances to become infected with SARS-CoV-2 infection includes obesity, hypertension, cancer, and diabetes. Cancer marks as a second major leading cause of death worldwide estimated with 10 million deaths in 2020. Majorly these patients will be more susceptible in contracting SARS-CoV-2 and will be more prone to develop severe and adverse conditions [3,11]. There are more chances for the cancer patients to contract SARS-CoV-2 as they have weak immune response that can make them sensitive to the virus (Figure 1). They are also susceptible to develop serious and fatal conditions include septic shock, acute respiratory distress syndrome (ARDS), and acute myocardial infarction [12,13,14]. The standard care of cancer treatment as well as against COVID-19 seems to be challenging now, it faces a puzzle between Cancer and COVID-19 crisis which one to deal first or deal with them together can solve the problem. The doctors are feeling pressurized of the exposure of their cancer patients against COVID-19 and how to maintain balance with suggesting them with an efficient therapy and guidance to their patients. Due to the COVID-19 healthcare, the cancer patients need to suffer which make them feel vulnerable as they need to be more cautious about their health and they need to seek guidance over telephonic conversation and avoid in-person meetings with their doctors [15].

Amalgamation of an immunotherapy with the nanotherapy could be a sign of relief for the cancer patients which can give a ray of hope and new direction to doctors to monitor their patients and give the best suitable treatment that could be the amalgamation of therapies rather than chemotherapy, radiotherapy, surgery, hormone therapy or targeted treatment alone. Elevated levels of interleukin-6 (IL-6) detected in the serum of the patients with severe acute respiratory stress associated with COVID-19 [16]. So, there is chance to use antibodies to block IL-6 or the receptor of IL-6 such as siltuximab (Sylvant TM EUSA Pharma), tocilizumab (ActemraTM Roche Genetech), sarilumab (KevzaraTM Regeneron), these are approved by FDA for certain conditions includes the rheumatological and lymphoproliferative disorders. These antibodies could be administered on immediate effect in case of emergency situations for the patients who are in critical stage leading to hypoxia [17]. In the study from Hubei province of China, shows elevation of cytokine levels includes the IL-2, IL-6, IL-7, IL-8, IL-17, TNFα, TNFβ, and in the inflammatory markers includes the ferritin, d-dimer, lactate dehydrogenase and there are other markers too implying towards systemic inflammatory response [18,19]. More studies have been conducted in the epicentre of the outbreak, Wuhan, China indicated that elevated C reactive protein (CRP) and IL-6 elevated levels could be a prognostic factor for the mortality [20,21].

Many research institutes and companies are trying very hard to bring out the best vaccines into the society with high efficacy rate and less mortality rate. Some of the vaccines authorized and approved by different countries are Covaxin (Bharat Biotech, ICMR, Hyderabad, India), Comirnaty (Pfizer BioNTech, Fosun Pharma, Manhattan, New York, NY, USA), Moderna (Moderna, BARDA, NIAID, Cambridge, MA, USA), CoronaVac (Sinovac, Beijing, China), Covid19 Vaccine AstraZeneca (BARDA, OWS, Cambridge, UK), Sputnik V (Gamaleya Research Institute, Acellena Contracr Drug Research and Development, Moscow, Russia). The nanomaterials are capable enough in the formulation of the vaccines that can enhance the immune resistance due to its petite size of a particle, high potency of loading the drug, surface charge and bio adhesive nature. The nanocarrier has implicit immune stimulatory ability and can modulate their structure according to the components of the vaccines and encapsulate it inside or attached outside their surface in order to work effectively with antigen presenting cells (APCs) [22,23].

As the whole world is under chaos due to the pandemic, there is still an urge to figure out the best and simple way to detect, diagnose and control the infection. Artificial Intelligence (AI) can open the windows which can efficiently track the spread of the SARS- COVID-19, single out the patients more prone to the virus and control it on time before leading to collapse. AI can definitely anticipate the mortality risk by analyzing the data from the patients and this technology can absolutely enhance planning and medication [24,25,26]. In this review study, we discuss about the two most important therapies for the treatment in cancer- involvement of immune checkpoint inhibitors and the nano- based vaccines which could improve the survival rate therefore, we hypothesize looking into today’s scenario about the SARS-COVID-19 pandemic, both the doctors and medical researchers are finding very difficult to out run the situation but if the amalgamation of an immunotherapy and nanotherapy is involved then definitely there is a chance to efficiently provide synergistic effect and furthermore adding to our strategy the use of AI which can reverse the whole situation for the cancer patients by tracking the patients infected with the virus at an early stage and the doctors could monitor their severity conditions and provide the best alternative or modification in their treatment regime. This would be an important task where both biological therapies plus the machine learning software work together in a very smart way.

## 2. Manifestation, Diagnosis and Management of SARS-CoV-2

**Figure 2:**
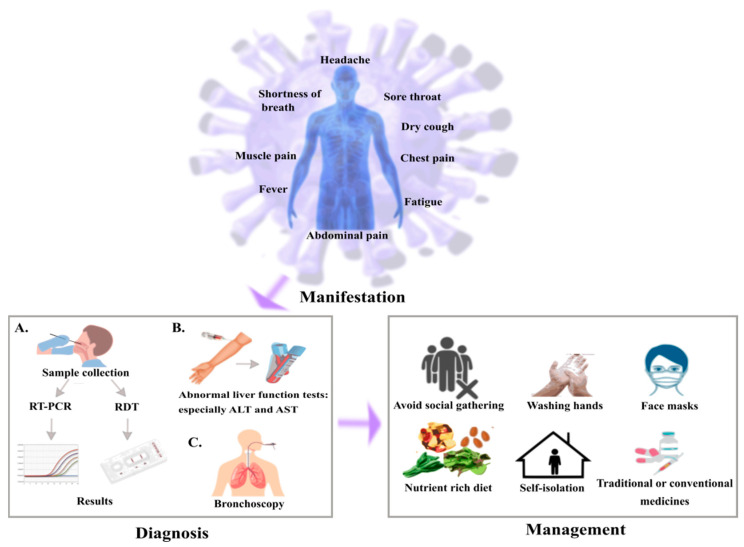
Illustration of scheme for SARS-CoV-2 in cancer patients. Common symptoms observed for the cancer patients with SARS-CoV-2 are fever, dry cough, fatigue, dyspnea. In diagnosis, (**A**) sample has been collected from throat or nasopharynx using sterile swabs followed by gene amplification and genetic analysis through RT-PCR or rapid diagnostic test (RDT), (**B**) abnormal liver function tests result in elevated levels of alanine aminotransferase (ALT) and aspartate aminotransferase (AST), (**C**) bronchoscopy is performed to avoid complications. In the management, cancer patients need to be more cautious compared to the normal people by restricting social meetings, maintaining good hygiene and self- isolation to avoid becoming infected and intake of medicines as prescribed by doctors.

The COVID-19 is spreading rapidly in all the countries irrespective of the rules and regulations imposed by the government to limit the infection. The mortality rates are higher in the elderly people, patients with multiple diseases/disorders and cancer patients [27,28]. It can be easily transmitted if a person is coughing or sneezing and later touches the eyes, nose or mouth with hands been laid on the surface of the droplets. On an average, the incubation period lies from 1–14 days with the median range of 5–6 days Bai et al., 2020 has reported case with incubation period of 24 days [29,30,31]. The outline of the SARS-CoV-2 in cancer patients has been demonstrated in the Figure 2 about the various symptoms that could be from mild to severe includes fever, dry cough, shortness of breath, headache, sore throat, abdominal pain, impaired consciousness, fatigue, nasal congestion, vomiting, diarrhea and there are also patients who doesn’t show any of the symptoms [31,32]. The rapid spread of COVID-19 makes difficult to determine the definite mortality rate of the infection at this stage. The major uncertainty factors susceptible to infection include the age, chronic lung diseases, congenital heart disease, hypertension, diabetes and cancer [1,18].

The common way to diagnose the infection is by the use of RT-PCR technique which requires nasal swabs, bronchoalevolar and tracheal aspirate but majorly nasal and oral swabs are commonly used for the diagnosis. Bronchoscopy isn’t usually recommended as a diagnostic approach because the aerosol possesses a huge risk both to patients as well as the hospital staff [33]. There are studies which states SARS-CoV-2 can also be detected in blood, stool and urine samples [19,34,35,36]. The RT- PCR method has very high specificity and less chance of false positive results which may be due to contamination of swab particularly in asymptomatic patients. So, the rate of sensitivity is predicted to be around 66–80% [37]. One negative result doesn’t omit the chances of SARS-CoV-2 infection, mostly in the cases of highly exposed persons just by taking a swab from the nasopharynx, in those cases it is recommended to take the test again or collect the specimen from the deeper region of respiratory tract by bronchoscopy [1,38].

**Figure 3:**
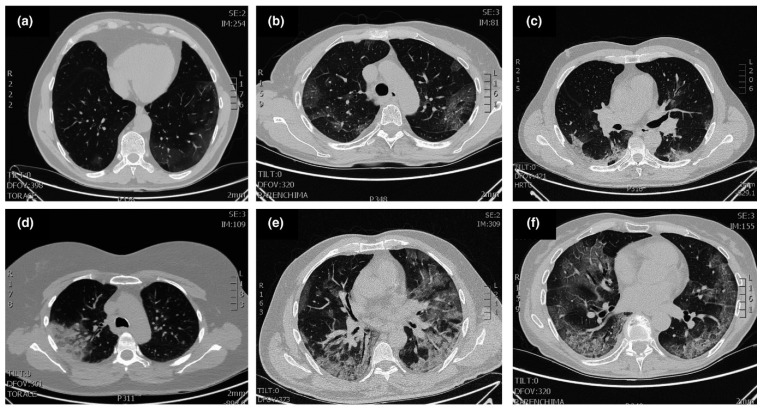
Typical patterns of COVID-19 at CT imaging. (**a**) Ground glass shadows (early stage), (**b**) Ground glass opacities, (**c**) Ground glass nodules and subpleural consolidation, (**d**) Focal consolidation, (**e**) Multifocal consolidation, (**f**) Multifocal consolidation with honeycomb (end stage). Reproduced with permission. Ref. [1] Journal of Internal Medicine © 2022 John Wiley and Sons, Inc.

**Figure 4:**
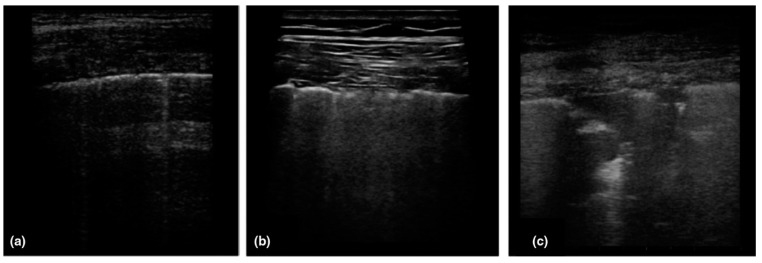
Patterns of COVID-19 at chest ultrasound. (**a**) Early bilateral multifocal areas of interstitial syndrome, (**b**) Interstitial pneumonia characterized by interstitial syndrome with B lines and preserved sliding sign, (**c**) Advanced, and organized pneumonia with interstitial syndrome associated with multiple subpleural consolidations and reduced sliding sign. Reproduced with permission. Ref. [1] Journal of Internal Medicine © 2022 John Wiley and Sons, Inc.

Some of the other clinical tests have been performed on the severely affected patients hospitalized and there are certain abnormalities with pneumonia includes lymphopenia (63%), leucopenia (9–25%), leucocytosis (24–30%), elevated levels of aspartate aminotransferase and alanine aminotransferase (37%) [36,39]. In a study of 1099 COVID patients, it has been found patients with lymphocytopenia (83%), thrombocytopenia (36%), leucopenia (34%) [40] and also seen in few patients with moderate hypertransaminasaemia, thrombocytopenia and elevated levels of lactate dehydrogenase [41]. Another way to diagnose the SARS-CoV-2 infection is performing CT scan of the patients and lungs will have ground glass opacities especially in lower and peripheral lobes and also seen in bilateral multiple lobular and sub-segmental regions for the patients admitted in ICU as depicted in Figure 3 [1,42,43]. According to the severity of SARS-CoV-2, the lung segments involved will vary and these opacities will become thicker with the advancement of the virus. In different studies, CT has higher sensitivity in patients with positive RT- PCR results approximately from 86–97% and lower in patients with mild symptoms approximately 50% [44]. The sensitivity for the typical chest X-ray is around 59% and an ultrasound has been used in few cases for diagnosis due to its low specificity and influenced by certain factors such as severity of virus, weight and proficiency of operator with sensitivity around 75% [41]. The progression of virus can be monitored and detected by interstitial appearances on the ultrasound of the patients as shown in Figure 4 [1,43].

Need of the hour to avoid SARS-CoV-2 infection is self-health-care management and isolation from the infected patients. The quarantine measures should be followed diligently and must isolate themselves if met symptomatic or asymptomatic patients [45]. Avoid social gathering, washing hands frequently, sanitize them, maintain social distancing, and wear masks whenever outside in crowded areas [46]. Keeping hydrated, having nutrition- rich diet and taking care of the mild symptoms such as fever, sore throat and dry cough with traditional home remedies or conventional drugs [47].

## 3. Risk to Cancer Patients of SARS-CoV-2 Infection and How to Monitor Them?

**Figure 5:**
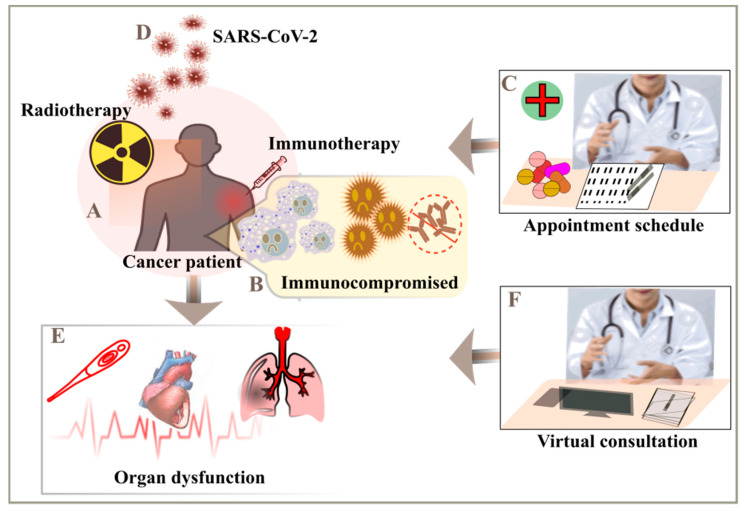
Monitoring of cancer patients to mitigate the severity risk. (**A**) Immunotherapy or radiotherapy considered for the treatment of cancer patients, (**B**) they usually have weak immune response, (**C**) follow-ups after the therapy, (**D**) cancer patients are more susceptible to SARS-CoV-2, (**E**) multiple complications includes septic shock, acute myocardial infarction (AMI) and acute respiratory distress syndrome (ARDS), (**F**) avoid visits to hospitals and opt for telecommunications.

The cancer patients are more prone and susceptible of developing the SARS-CoV-2 infection which makes them into being vulnerable. Cancer makes the patient fall into an immunosuppressive condition due to various treatments with therapies such as chemotherapy or radiotherapy [48]. Due to weaken immune response, if the cancer patients are infected with SARS-CoV-2 they may be potent of developing serious and fatal conditions such as include septic shock, acute respiratory distress syndrome (ARDS), and acute myocardial infarction [12,13,19]. The care for the cancer patients with the COVID-19 seems similar to a challenge on both the swings, due to arrangement of resources and managing the emergency situations against COVID-19. The doctors and the frontline workers are in a state of plight, how to make balance of the COVID-19 risk with the providing an efficient therapy and guidance to their patients. On looking over to another perspective, the cancer patients is certainly affected by the COVID-19 as they are in a vulnerable state and for their follow-ups, they need to avoid in-person meetings at hospitals and follow their treatment over a virtual platform. They are majorly suffering due to this pandemic, as they need to cancel their routinely appointments and reschedule it for later (Figure 5) [15].

Liang et al., 2020 have reported the cancer patients infected with SARS-CoV-2 infection are at higher risk of serious complications compared to patients without cancer. The death rate was also increased in the cancer patients with COVID-19 infection, so it would be beneficial for the patients if they can postpone their chemotherapy sessions or surgery but in the case of emergency, personal protection equipment and strict protective measures need to be followed to avoid transmission of the infection [49]. The elderly patients of non-small cell lung cancer with the age of over 60 years had higher chances of becoming infected with SARS-CoV-2 infection compared to the younger patients. According to the studies, it can be assumed to limit the number of hospital visits to becoming prone to catch the infection, staying at home and following the treatment at your own premises could be considered as the best option [50]. Zhang et al., 2020 also found high mortality rate in the tumor associated cancer patients compared to the non- tumor cancer patients. On the other hand, receiving the anti- cancer treatment within a period of 14 days of an infection led to increased risk of associated with severe symptoms [51]. Dai et al., 2020 took into an account of 536 normal patients and 105 cancer patients infected with SARS-CoV-2 from Hubei, China and they found out the cancer patients were more prone to severe symptoms of COVID-19. The high death rate and severe symptoms were observed in blood cancer and lung cancer at metastatic stage. The anticancer treatments has been provided but it led to developing severe symptoms which demanded to admit the patients in ICU and under ventilation [52]. In the clinical study performed in New York, where a total of 2035 patients were enrolled, out of which 423 were cancer patients infected with SARS-CoV-2. 40% of the patients were hospitalized, 20% contracted severe COVID-19 and 12% of them died. The predicted factors for the cause were demand of oxygen supply, ICU, age, sex, previously or ongoing anti-cancer treatment [53].

Many guidelines and protocols have been forwarded to the hospital staff by European Society for Medical Oncology (ESMO), American Society of Clinical Oncology (ASCO), Italian Society Association of Medical Oncology (AIOM), in regard, for the safety, care, good health management, and avoid chances of transmission in the cancer patients. The doctors are allowed to follow up their patients over a virtual call and avoid unneeded attention in the transmission of virus at the centers/hospitals [54,55]. The utmost priority is that cancer patients spend the least time in the hospital and recommended to follow oral or subcutaneous mode of administration rather than intravenous and to cancel surgery when not an emergency [56,57].

## 4. How Can Blockage by Immune Checkpoint Inhibitors Be Beneficial for the Cancer Patients?

**Figure 6:**
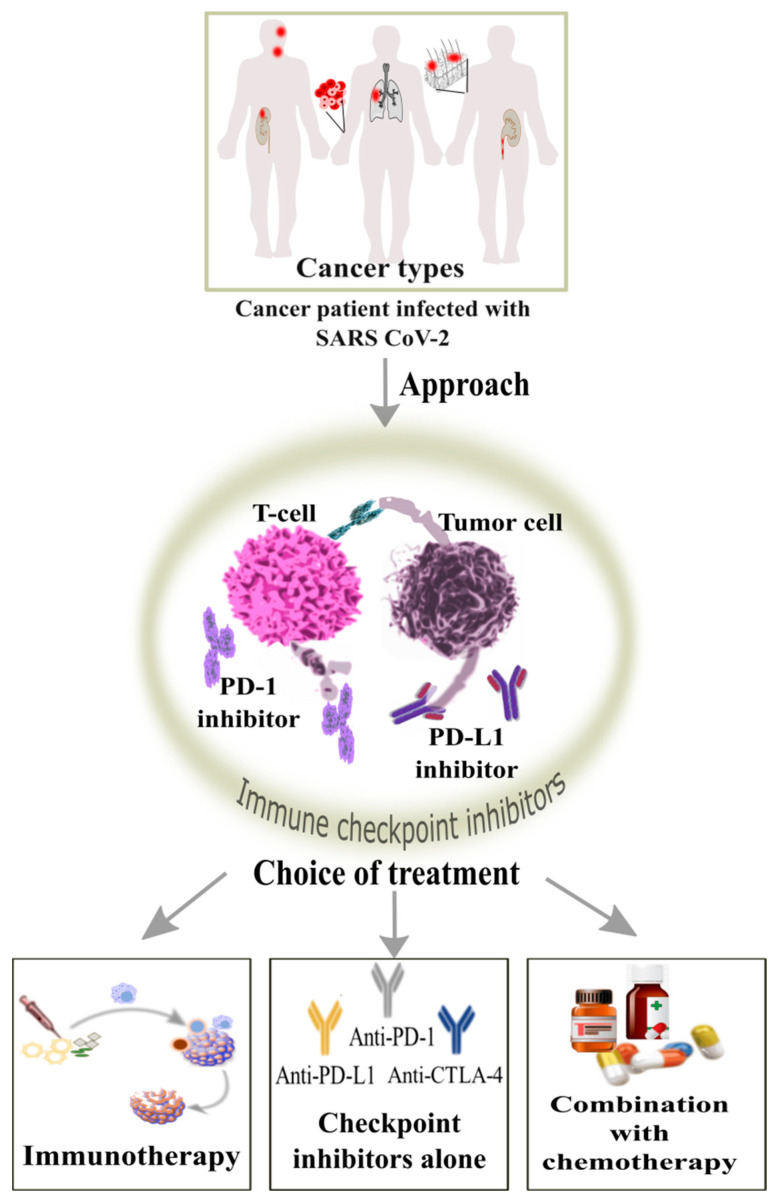
Role of action by immune checkpoint inhibitors for blockage cancer patients having solid tumors infected with SARS-CoV-2 can opt for the treatment by the use of immune checkpoint inhibitors such as anti-PD-1, anti-PD-L1 and anti-CTLA-4 antibodies which can increase the CD8+ T cell response and viral clearance.

The immune checkpoint inhibitors (ICIs) have transformed from time to time with the management of the cancer. ICIs are usually the monoclonal antibodies aimed to work against the immune checkpoints which are activated by the cancer cells to silence the immune response and its anti-cancer role [58,59]. They include an anti-PD-1, anti-PD-L1 and anti-CTLA-4 antibody which shows a progressive immunotherapy treatment of solid tumors includes the head and neck, urothelial, melanoma, haematological, renal carcinoma and lung cancer, it could be alone, in combination or with chemotherapy [60,61,62]. The anti-tumor cellular mediated immune response is recovered, and the cancer cells could be removed from the immune system by the blockage of PD-1/PD-L1 and CTLA4/B7 checkpoints (Figure 6) [63].

Souza et al., 2020 showed that two metastatic melanoma patients were treated in combination with anti-PD-1 and anti-CTLA-4, later the patients had side effect during the treatment and developed pneumonitis but were tested negative against SARS-CoV-2 infection. Even though they were monitored for a week, but they were negative for the virus infection, but cancer patients are very sensitive to ICIs [64]. There was another case study on the two cancer patients infected with SARS-CoV-2 infection were treated with ICIs, they had mild symptoms and recovered from the virus infection. It seems that this treatment can be taken into consideration for the cancer patients with COVID-19 infection as they can be recovered with less severe symptoms [65]. In the study, reported that there was a significant progress observed in the cancer prognosis treated with ICIs and it should be administered regularly to see the improvement [66]. The list of immune checkpoint inhibitors involving PD-1, PD-L1 and CTLA-4 are enlisted in the Table 1 including ongoing as well as completed clinical trials with a range of different cancer types. One clinical study is ongoing and will be accessible till the end of current pandemic; they have opened a register named TERAVOLT, where they are collecting data for thoracic cancer patients contracted with SARS-CoV-2 infection. According to the preliminary data, it shows that ICI therapy doesn’t increase the overall mortality rate for the cancer patients infected with virus infection nor their frequent hospitalization visits [12].

Due to this pandemic, there are different scientific approach forecasted by different researchers/groups but till now no conclusive data has been found out. It has been found that there is no confirmation regarding the toxicity of the ICI therapy or not and whether there is an increase in the risk to the cancer patients contracted with the viral infection. Recently, most of the cancer care centres are practicing ICIs specifically for the lung cancer patients [56,67].

The cytokine release syndrome triggered by the cytokine storm leads to serious complications in the patients infected with COVID-19 and it can cause ARDS [68]. It has been observed that both CD4+ and CD8+ T cells start to differentiate in the patients with severe SARS-CoV-2 infection and those cells will be activated less and reduced in cell number. The viral clearance is slow due to the extreme depletion of CD8+ T cells in the severely infected COVID-19 patients which may lead to less cellular-mediated immune response against SARS-CoV-2 virus [69,70]. And ultimately, the severely affected COVID-19 patients will develop viral septicaemia [71].

Barber et al.; Day et al., 2006 have validated PD-1/PD-L1 blocking antibodies conducted on mice and humans persistently infected with viruses, it was interpreted that it enhanced the viral clearance and CD8+ T cell response. This makes PD-1 blockade more important to regain the lost T-cells formation and both CD4+ and CD8+ T cells can be recovered in number as well as their functionality [72,73]. In a recent study, they involved lung cancer patients with COVID-19 to find out the PD-1 blockade in the severely affected patients, where few patients already received the PD-1 blockade, so they divided into four categories: received PD-1 blockade, received PD-1 blockade within last 6 months, received PD-1 blockade within last 6 weeks, received within last 3 weeks. They found out that there was no significant relationship between the PD-1 blockade and severely affected COVID- 19 patients. Further to evaluate the potency of their results, they monitored the close relationship of PD-1 blockade exposed to evaluate the COVID-19. With the increase in severity, they examined and found previous history of smoking creates an imbalance, *p* < 0.001 received PD-1 blockade before versus no PD-1 blockade and it creates a high chance to hit severity in COVID-19 patients, most probably associated with pulmonary dysfunction and other comorbidities [13,74,75,76,77]. With the alteration in smoking status, change in ORS to monitor the effect of PD-1 blockade on ICU/intubation or hospitalization or death rate all reduced to nearly 1. With these reports, it can be said that PD-1 blockade can be safe to be used for the cancer patients [78].

## 5. Therapeutic Strategy to Mitigate the Stress on the Healthcare

In the current era, the healthcare system has been burdened on how to attenuate SARS-CoV-2 and their patients with the best suitable treatment and care. It has been found that there is severe onset of the virus and detrimental changes in the immune system which is commonly known as “cytokine storm” involving the elevation in the levels of IFN-γ, IL-8, IL-6, IL-1β, and TNF-α. With this pro-inflammatory release of the cytokines has been associated with poor survival rate as well as severe lung complications [39,79]. The therapeutic strategy by IL-6, IL-1 or TNF-α pathways which could halt or balance the systemic inflammatory response syndrome (SIRS) or the other such complications [80]. In many cases, it has been observed that there are elevated levels of C reactive protein (CRP) and aspartate transaminase, the report from the China also shows that inflammatory markers include ferritin, d-dimer and lactate dehydrogenase have shown elevated levels [18]. Ruan et al., 2020 involved 150 patients from Wuhan, China stated that elevated CRP and IL-6 levels were the prognostic signs of mortality, as 68 patients who died had a median level of IL-6 about 11.4 ng/mL compared to the 82 patients who survived had about 6.8 ng/mL. They also mentioned that elevation in the ratio of the neutrophil and leukocyte showed poor survival rate [20].

### 5.1. How Immune Cells and Their Targets Are Helpful for the Treatment?

In many of the countries, the healthcare system is facing one of the difficult times due to shortage of resources including the ICU beds, ventilators, oxygen supplies and many more. With this scenario, maintaining social distancing and treating their patients is quite challenging as they weren’t prepared for the huge number of patients coming in with severe cases and sometimes, they need to make the decision between patients. They are working very hard to reduce the number of patients with severe complications and the time required on the ICU bed or the ventilator. It has become very important to treat the infected individuals to reduce the load on the healthcare system. So, the regulation or the inhibition of IL-6 could be beneficial to suppress the inflammatory response in the individuals. This approach has been followed for the patients engaged with T-cell treatment including CAR-T cells or monoclonal antibody, blinatumomab. As the levels of IL-6 rise at a period of time with maximum proliferation of T cells and then patients tend to develop cytokine release syndrome which could be very serious and uncompromising. There are many IL-6 inhibitory drugs available to reverse the action of cytokine release syndrome in the patients such as siltuximab and tocilizumab [81,82].

The studies from the China and Italy supported the use of tocilizumab which helped in the clinical intervention with decline in the inflammatory response, pulmonary problems and showed improvements in the patients with critical COVID-19 response. The phase II/III clinical trials studies have been entered since 2020 to mark the safety and efficacy of the tocilizumab (NCT04320615) for the severely ill patients with pneumonia. Another phase II/III clinical trial to assess the efficacy of sarilumab (NCT04315298) is undergoing. As the studies from China have shown the recovery rate was higher for the severely ill patients treated with tocilizumab and the symptoms disappear even before the start of the cytokine storm. It has been suggested to monitor the patients closely by performing CT scans or checking the increased levels of the CRP and IL-6 as the condition can change from mild to severe and early treatment is recommended. Currently, the most promising anti-IL-6 agents are tocilizumab and sarilumab but certainly many other agents are also in the pipeline to alter the pro-inflammatory response and help to flatten the curve for the hospitalized patients.

There are other inhibitors which can also be thought for the treatment or prevention of the cytokine release such as JAK inhibitors—tofacitinib, baricitinib, ruxolitinib, peficitinib, upadacitinib and fedratanib, IL-1 inhibitors—anakinra, rilonacept and canakinumab, TNF-α inhibitors—etanercept, adalimumab, certulizumab, golimumab and infliximab, IFN-γ inhibitor—emapalumab, GM-CSF inhibitors—namilumab, gimsilumab, lenzilumab, TJM-2 and otilimab, IL-17 and IL-23 inhibitors—brodalumab, secukinumab, ixekizumab, ustekinumab, guselkumab, tidrakizumab and risankizumab [21]. However, these inhibitors need to be explored more for their safety and efficacy whether they could be administered into the cancer patients with elevated serum levels and cytokines.

### 5.2. How Nanocarriers Beneficial and Involved into the Treatment Regimen?

There are many vaccines, drugs and new molecules been rolled out in the market but still there is an urge to monitor and be promising for the treatment of the COVID-19 cancer patients. So, another direction could be nanocarriers based therapeutics in the coming future to control the pandemic, as nanotherapy has turned out to be very fruitful against various diseases and there is still a hope that it can also overcome the SARS-CoV-2 outbreak [7]. The nanocarriers could be beneficial for the prevention, diagnosis and treatment which can interfere with the immunological response of the body. The nanocarrier systems have been monitored for its efficacy and they have entered into preclinical studies against different viruses includes human papilloma virus, herpes simplex, HIV and many more [83,84].

The nanocarrier delivery system needs to be very precise and accurate for its efficiency and safer use. The delivery of the medication involving the protein, peptide or DNA/RNA to the respective target site is usually hindered because of the reduced bioavailability, degradation, clearance out of site and its solubility which leads to the lower therapeutic actions. The challenges of the administration of traditional therapeutics will involve the large surface area to volume ratio, surface modification with different functional groups, and different physicochemical properties of the nanomaterials. The organic as well as inorganic nanomaterials can be enclosed with abundant load, to control the release of the drug, reduce drug resistance, and improve the pharmacological effect of the drug on the patient. The active targeted nanocarriers can reach out to the respective target sites by crossing the biological barriers with high specificity and avoid risk to the non-targeted sites by reducing dosage, toxicity with enhanced efficacy and bioavailability [85,86]. From the previous studies, encapsulation of acyclovir in chitosan nanocarrier has been used for the treatment of herpes simplex virus, Ref. [87] lipid encapsulated silica nanocarrier was used for the delivery of the antiviral compound to inhibit the viral action of encephalitis virus which enhanced its stability and solubility as well as it increased the circulation time and biocompatibility, Ref. [88] encapsulation of liposomes with cholesterol and modified with the drug, hydroxychloroquine for the treatment of pulmonary fibrosis [89]. It has been observed that encapsulating the nanocarrier offers stable nature for the DNA/RNA based vaccine which helps the transport of genetic material inside the system to the cells. The vaccines with siRNA/mRNA can be encapsulated with lipid or the polymer nanocarriers because of their ion charges and can conjugate with the nucleic acids for the translation of the genetic material. This treatment regime offers a great platform for the inhibition of the virus with the modification of the nanocarrriers to treat the viral infections [90]. The lipid-based nanoparticle vaccine; mRNA-1273 has passed the clinical trials and shows the 94.1% efficacy in a two-dose regimen [91].

The combination therapy involving the nanocarriers based approach provides a synergistic action, which can open another therapeutic window for the treatment of SARS-CoV-2. It offers reduced amount of dosage of treatment regime in order to avoid any severe side effects and also combat drug resistance. So far, nanocarriers have been proved beneficial for the delivery of the drugs with their respective physiochemical properties. It is also believed that nanocarriers could be beneficial for the multidrug approach to control the inflammation and the prognosis of the virus. Freeling et al., 2014 have been involved with the multidrug approach by nanocarriers, a lipid modulated multi-drug nanoparticle involving anti-retroviral drugs such as ritonavir, lopinavir and tenofovir to overpower the inadequate delivery of the drug to the lymphatic system, which stated increase by 50-fold and maintained intracellular concentrations inside the lymph nodes in comparison to the oral delivery of the drugs [92]. As the new variants of SARS-CoV-2 are becoming threat to the humanity, the divergence of antigenic protein in the nanocarrier system could strengthen the potency of vaccines against the viruses. This vaccine approach can be incorporated to protect against the degradation of antigenic proteins as well as enhanced exposure to antigen presenting cells to increase the potency and delivery of the drug.

The nanocarriers could serve the purpose of both the stimulation as well as suppression of the immune response wheresoever necessary. The disadvantages of the traditional-based vaccines involves the short half-life and absorption rate, less stability in blood, poor immune response, side effects due to high dosage, non-specific and require large storage conditions. On the other hand, nano-based vaccines have adjustable size and surface properties, active immune stimulation benefits, good humoral as well as cellular responses, targets at multiple areas and have less severe side effects [93].

The use of nano-materials as the vaccine adjuvants can enhance the potency of vaccine due to lower toxicity, active target sites, high surface area and low antibody titre value [93,94]. Vaccine adjuvants-based nanoparticles are designed to boost its safety and potency inside the immune microenvironment [86,95]. Many of the pre-clinical trials are undergoing for the COVID-19 vaccines and many of them involve the combination of antigen and adjuvant. One of the nanoparticle-based vaccine combined with the adjuvant as matrix M; NVX-CoV2373 has passed the clinical trials and shows 89.7% of the efficacy against SARS-CoV-2 infection in a two-dose regimen [96]. These vaccines are being designed to guide the immune cells against an antigen and provide protection by the immune system to improve its effectiveness against the danger-associated signals [97]. These adjuvants can provide counter-act signals to the immune system to be tolerant against the foreign antigens. And the adjuvant-based nanocarriers can develop physico-chemical properties for the stimulation against the pro or the anti- immune pathways [98].

As the administration of adjuvants in the inactivated and recombinant based vaccines is carried out to increase its immunogenicity, so the nanocarriers based on their functionality acquires an inherent adjuvant site to enhance the host’s immune response. This inherent site could be favourable for the manipulation of the nanocarriers to enhance host’s immune response specifically for damaged immune system patients, as the nanocarriers could strengthen the delivery of immunosuppressants [99].

There are few metallic and non-metallic nanoparticles such as silver, copper, gold, zinc and graphene oxide which have inherent anti-bacterial as well as anti-viral properties to serve as a therapeutic target. Nanoparticles based approach can control the drug release, increase the entry inside the cell and reduce toxicity from ions. The metallic nanoparticles are highly preferred as the antiviral agents due to its smaller chance of drug resistance. From the studies, it has been found that silver nanoparticles show antiviral action against HSV, HIV, Hepatitis B virus and many more [86,100]. And they bring less toxicity and are harmless to humans at lower concentrations which serve as a therapeutic option. It has also been reported that nanocolloidal silver nanoparticles turns out to be efficient against the viral proliferation in the respiratory tract after the delivery through inhalation, thereby reducing the progression of virus [101].

The nanotechnology could be helpful in the early detection, monitoring and elimination of viruses; for the detection, the nanoparticle-based approach will stimulate the patient’s health management, identification and regulation of the infected hotspots; the metallic nanoparticles can monitor the virus via the magnetic, fluorescence and surface enhanced raman scattering which improves to track down response from the treatment [86,102]. One study stated that graphene sheets bound to antibodies can easily help in the detection of viral proteins and this can serve as biosensors against SARS-CoV-2 [103]. The nanomedicine approach could be one of the options where both the diagnosis and treatment can be targeted together for the detection and neutralization of the virus, thereby causing reproducibility and growth hindrance of virus. Since the outbreak of SARS-CoV-2, many of the nanomedicine based targeted approach candidates are under the surveillance [104].

## 6. Looking into Another Perspective: Artificial Intelligence Opening Gateways against SARS-CoV-2

As this pandemic has created a huge impact and challenged the medical facilities worldwide and there is an urgent need to diagnose the patients infected with COVID-19, especially with the cancer as they are more vulnerable at this stage due to their weakened immune response. Artificial Intelligence (AI) is one of the technologies which can help to mitigate the spread of the virus by tracking down the patients at higher risk of infection and their mortality rate. AI workflow involves the collection of data; preparation of data; learning algorithm and training; testing; prediction and analysis. The data of the cancer patients infected with or without COVID-19 will be collected and prepared according to different characteristics such as age, sex, gender, cancer type or stage, smoking or drinking, cytokines level, previous treatments, and other common symptoms. In the learning process, the system will be trained accordingly with the respective characteristics; testing will make sure about the system’s efficiency and performance. Finally, the prediction and analysis will be carried out by the system to predict the severity of cancer patients with a particular type of cancer and their respective stages.

**Figure 7:**
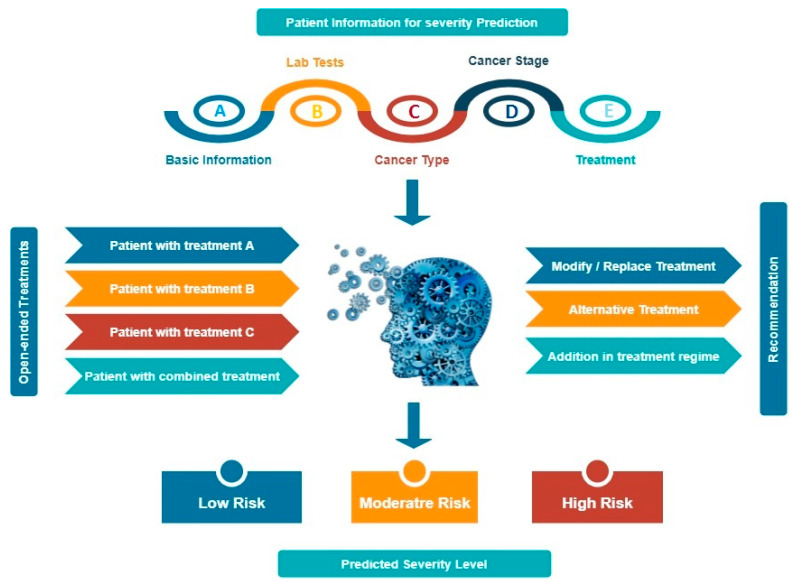
Incorporation of machine learning algorithm for the prediction and analysis On the top and left panel involves the input data whereas on the bottom and right panel involves the output data after the training of machine. The topmost input data involving (**A**) basic information—age, sex, race and common symptoms, (**B**) lab tests—WBCs, lymphocyte, neutrophil, platelet count, C-reactive protein level, lactate dehydrogenase, D-dimer level, IL-6 level and so on, (**C**) cancer type—lung, breast, prostate, gastric, ovarian, leukemia or others, (**D**) cancer stage—I, II, III, IV, (**E**) treatment—radiotherapy, chemotherapy, immunotherapy, surgery or targeted therapy or others. The output data can help in the prediction of the severity level—low, moderate or high. The left input data involves the ongoing treatments of the patients could be radiotherapy, chemotherapy, immunotherapy and the output data could help the oncologists to recommend their patients; whether the treatment needs to be modified, altered or additional changes required.

In the current review paper, we hypothesize an idea for the future by the involvement of AI and it would become easier to make prediction beforehand and make analysis through various parameters (Figure 7). As we were not able to acquire the clinical data from hospitals directly, so we couldn’t train the machine for a small dataset. Our ideology says, if the input data is fed into the machine involving the patient’s information—age, sex, race, common symptoms; laboratory findings includes WBCs, neutrophils, lymphocytes, monocytes, eosinophils, platelet count, haemoglobin, alanine aminotransferase (ALA), aspartate aminotransferase (AAT), serum creatinine and lactate dehydrogenase (LDH) levels, CD4+ and CD8+ T cells, B lymphocytes, IL-2, IL-4, IL-6, IL-10, TNF-α and TNF-γ levels; cancer types involving lung, breast, prostate, ovarian, gastric or others, different four stages of cancer involved; past/ongoing treatments could be radiotherapy, chemotherapy, immunotherapy, targeted therapy. This data would help the physicians to predict the severity level of the virus in the cancer patients and they can put them in the priority list for the immediate treatment. On the other hand, if the input data is fed about the undergoing treatments by the patients could be radiotherapy, chemotherapy, immunotherapy or targeted therapy could have the output results for the physicians to provide best recommendations to their patients about the modification, alteration or addition to the undergoing treatment.

There are many applications of AI during this pandemic involving the detection and diagnosis which could help in the early stage of infection and help the physicians to take decisions wisely. With this technology, many new algorithms can be trained for the early diagnosis of the infection and the evaluation of treatment with such kind of machine learning approach; it becomes easier to predict the spread of infection and change the treatment of patient if required according to daily updates. It can help to trace the hotspots of infection and track the patients infected in clusters. The mortality rate can be predicted for the people who are more vulnerable to the virus whether they belong to different regions, caste, religion, or countries. AI could also be beneficial for the drug and vaccine development, to check the testing of the drug in real time compared to the conventional methods which takes longer time. The burden on the healthcare would be reduced by monitoring the cancer patients through machine learning and provide the best treatment to the infected patients at an early stage. The progression of cancer could be prevented by analyzing the data in real time and monitor the infections at different time intervals which could avoid any epidemic or pandemic in future [25].

## 7. Conclusions

With the high number of people becoming infected, the healthcare system is burdened to provide the adequate facilities to each patient as the resources are not enough which puts a lot of pressure on the medical workers. From the previous studies, it is evident that cancer patients infected with SARS-CoV-2 are more vulnerable and prone to develop severe infections than compared to the patients without cancer due to their weakened immune response because of chemotherapy or radiotherapy sessions received and they can easily become exposed to the infection with the severe outcomes. The patients with lung, breast, ovarian, leukemia, gastric cancer, and those in third or fourth stage of cancer have shown severe symptoms and higher mortality rate. The cancer patients are being asked to postpone and schedule their appointments for later or if possible, to ask for advice via telecommunication or video calls. In addition, there is an immediate urgency to look from a different perspective to combat and win against this infection and reduce the mortality rate across the world. In this review, we tried to propose an idea about the incorporation of combined treatment regimens for the cancer patients infected with SARS-CoV-2 and how to predict and analyze the real time data to avoid the severity of the infection in other patients. The use of immunotherapy by the different immune checkpoint inhibitors are undergoing clinical trials for its safety and efficacy as well as CAR- T cell therapy whereas on the other hand, nanotherapy involves the different types of nanoparticles, aiming at a specific target site and modify the design by the encapsulation of particle. These combinations of therapies could serve best for the preparation of vaccines for the immune-compromised patients with a smaller number of side effects as the release can be controlled at the specific targeted site with the adequate amount of dosage. However, there is also a crucial need to avoid the severe outcomes in the other patients with the similar case history. Artificial intelligence could help us to access the real time data of the people which serves as the icing on the cake to avoid any severe or critical conditions in the future. The use of prediction models that involves several features to estimate the risk of infection, could provide assistance to physicians to change or provide alternate treatment for their patients. The effective screening will help in immediate and competent diagnosis of the cancer patients infected with SARS-CoV-2 to minimize the burden on the healthcare. In the future, if possible, to acquire the clinical data of cancer patients from the different hospitals the severity of risk and severe outcomes could be predicted using real time data.

## Figures and Tables

**Table 1:** Immune checkpoint inhibitors ongoing and completed clinical trials.

Immune Checkpoint Inhibitor	Target Type	Clinical Trial Identifier	Sponsors/Developers	Against the Type of Cancer
Pembrolizumab	PD-1	NCT01295827, NCT02658019, NCT04533451	Merck Sharp and Dohme Corp.; Lynn Feun, MD, University of Miami; Alliance for Clinical Trials in Oncology	Melanoma, NSCLC, Hepatocellular carcinoma
Nivolumab	PD-1	NCT01844505, NCT00730639, NCT01928394	Bristol-Myers Squibb	Metastatic melanoma, Metastatic Castration-resistant Prostrate Cancer, Renal Cell Carcinoma, Non-small Cell Lung Cancer
Cemiplimab	PD-1	NCT04722523, NCT04154943, NCT04428671, NCT04050436	Memorial Sloan Kettering Cancer Center; Regeneron Pharmaceuticals; Emory University; Replimune Inc.	Head and Neck, Squamous Cell Carcinoma (HNSCC), Advanced Cutaneous Squamous Cell Carcinoma, Metastatic Skin Squamous Cell Carcinoma
Atezolizumab	PD-L1	NCT02031458, NCT02848651, NCT04776447	Hoffmann-La Roche; Genentech, Inc.; Fundación GECP	Non-Small Cell Lung Cancer
Avelumab	PD-L1	NCT01772004, NCT02603432, NCT02952586, NCT02155647	EMD Serono Research and Development Institute, Inc.; Pfizer	NSCLC, metastatic breast cancer, colorectal cancer, urothelial carcinoma, mesothelioma, gastric cancer, ovarian cancer, renal cell carcinoma, melanoma, head, neck squamous cell carcinoma, castrate-resistant prostate cancer, adrenocortical carcinoma
Durvalumab	PD-L1	NCT03043872, NCT02904954, NCT04774380, NCT04745689	AstraZeneca; Weill Medical College of Cornell University	Non-Small-Cell Lung carcinoma
Ipilimumab	CTLA-4	NCT01515189, NCT01844505	Bristol-Myers Squibb	Metastatic Melanoma

## Abbreviations

AI	Artificial Intelligence
SARS-CoV-2	Severe acute respiratory syndrome associated coronavirus
CSSE	COVID-19 Data Repository by the Center for Systems Science and Engineering
MERS	Middle East respiratory syndrome
ARDS	Acute respiratory distress syndrome
ACE2	Angiotensin-converting enzyme II receptor
AT1R	Angiotensin type 1 receptor
IL-6	Interleukin-6
CRP	C reactive protein
APCs	Antigen presenting cells
RDT	Rapid diagnostic test
ALT	Alanine aminotransferase
AST	Aspartate aminotransferase
LDH	Lactate dehydrogenase
ESMO	European Society for Medical Oncology
ASCO	American Society of Clinical Oncology
AIOM	Italian Society Association of Medical Oncology
ICI	Immune checkpoint inhibitors
